# Migration and mobility of third-country national labour workers to and inside Europe during the Covid-19 pandemic – a legal analysis

**DOI:** 10.1186/s40878-021-00229-1

**Published:** 2021-05-27

**Authors:** Adolfo Sommarribas, Birte Nienaber

**Affiliations:** Université du Luxembourg, 11 Porte des Sciences, L-4366 Esch/Belval, Luxembourg

**Keywords:** Legal migration, Legal analysis, European Union, TCNs, Covid-19, Labour market

## Abstract

The Covid-19 pandemic took most EU Member States of the European Union by surprise, as they underestimated the rapid spread of the contagion across the continent. The response of the EU Member States was asymmetrical, individualistic and significantly slow. The first measures taken were to close down the internal borders. The response of the European Union was even slower, and it was not until 17th March 2020 that the external borders were closed. These actions affected legal migration into the European Union from four perspectives: it affected 1) the mobility of those third-country nationals who were on a temporary stay in the EU Member States; 2) the entry of third-country nationals to do seasonal work; 3) legal migrants entering and staying; and 4) the status of the third-country nationals already residing in the EU Member States, especially those experiencing a loss of income. This article will deal with the measures taken by the EU Member States to manage the immigration services, as a case study how Luxembourg dealt to avoid that temporary staying migrants and regular migrants fall into irregularity. Finally, we will focus on the vulnerability of third-country nationals with the rising risk of unemployment and the risk of being returned to their country of origin. The article will also analyse access to healthcare and unemployment benefits.

## Introduction

There is a paradigm when labour migration is discussed: on the one hand, the European population is ageing (“Skilled Migrants Would Have It Easier to Move to EU If New Migration Pact Is Approved” 2020, pointed out by EU Commissioner for Home Affairs Ylva Johansson when presenting the New Pact on Migration), requiring migrants to cover the positions that cannot be covered by EU citizens, and on the other hand, local unemployment remains relatively high in the EU-27.

Goldner Lang (2018, pp. 8-9) states that the EU regulations provide for mobility for “desirable” migrants (especially the highly qualified). Labour migration remains one of the legal channels for migrants to enter the European Union, but their rights inside the EU depend on their status. Possible statuses for labour migrants at the EU level can be: highly qualified workers (Blue Card Holders), salaried workers, independent workers, ICT (intercorporate transferees), researchers, posted workers and seasonal workers according to these Directives: the EU Blue Card Directive (Council Directive 2009/50/EC of 25th May 2009 on the conditions of entry and residence of third-country nationals for the purposes of highly qualified employment), the Single Permit Directive (Directive 2011/98/EU of the European Parliament and of the Council of 13th December 2011 on a single application procedure for a single permit for third-country nationals to reside and work in the territory of a Member State and on a common set of rights for third-country workers legally residing in a Member State), the ICT Directive (Directive 2014/66/EU of the European Parliament and of the Council of 15th May 2014 on the conditions of entry and residence of third-country nationals in the framework of an intra-corporate transfer), the Directive on students and researchers (Directive (EU) 2016/801 of the European Parliament and of the Council of 11th May 2016 on the conditions of entry and residence of third-country nationals for the purposes of research, studies, training, voluntary service, pupil exchange schemes or educational projects and au pairing), and the Directive on Seasonal Workers (Directive 2014/36/EU of the European Parliament and of the Council of 26th February 2014 on the conditions of entry and stay of third-country nationals for the purpose of employment as seasonal workers).

At the national level, however, migrants’ access to the labour market is implemented, determined and controlled by national legislation (Goldner Lang 2018), but by entering the European Union via the different status for third-country nationals (TCNs), they do not obtain the same rights as EU citizens regarding e.g.

- restrictions on freedom of movement (a holder of a residence permit in a Member State is allowed to travel and stay in another Member State for up to 90 days in a six-month period, but they are not allowed to work during their stay or apply for permanent residence in the other Member State),
- access to labour markets (in most EU Member states, TCNs are obliged to pass a labour market test in order to access the labour market, giving priority to EU citizens and third-country nationals who are already resident in applications for vacant positions),
- access to unemployment benefits (in most EU Member States, access to unemployment benefits does not depend on nationality but mainly on age and contributions to the insurance scheme, although the length of time for which someone could benefit from them will depend on their residence status),
- access to healthcare (access to the scheme will depend on whether it is a public contributory scheme (i.e. Luxembourg (European Migration Network 2014)) or a private health insurance scheme to which the TCN will have to subscribe),
- change of status (some EU Member States are flexible with regard to the possibility of changing status between categories, but there are some categories of economic migrants that cannot change – i.e. seasonal workers in some EU Member States (European Migration Network 2015, p. 21), or
- equality of treatment (in principle, labour laws treat all the workers in the same way across the EU, but labour and immigration legislation provides for specific conditions that TCNs must fulfil in order to reside in the territory, i.e. work in a specific sector, renew their residence permit at precise intervals, undergo labour market tests, and there is the difficulty of accreditation of job qualifications/assessment of skills of TCNs, language barriers (European Migration Network 2019, p. 5) etc.).

Although the EU offers its citizens freedom of movement and access to the labour market, this looks different for third-country nationals. Article 7 of the Schengen Agreement (The Schengen acquis - Agreement between the Governments of the States of the Benelux Economic Union, the Federal Republic of Germany and the French Republic on the gradual abolition of checks at their common borders) of 14th June 1985 provides for the right of intra-EU mobility for TCNs under special conditions (uniform visa policy between the EU Member States to avoid adverse consequences in the field of migration and security due to the easing of border controls). These limitations were later reinforced by article 13 (2) of the Convention implementing the Schengen Agreement of 14th June 1985 between the Governments of the States of the Benelux Economic Union, the Federal Republic of Germany and the French Republic on the gradual abolition of checks at their common borders of 19th June 1990. Della Torre and De Lange (2018) show the integration of TCNs through freedom of movement, but also that they are confronted with “spatial temporal waiting zones” because of the legal framework. Others have discussed the different rights for TCNs as well (e.g. the restrictions on the freedom of movement (i.e. Goldner Lang 2018; Bazylińska-Nagler 2019; Della Torre and De Lange 2018), access to labour markets (Janda 2017; Verschueren 2018), access to unemployment benefits (Verschueren 2018), access to healthcare (Caterina Francesca and Petretto 2019; Koutsampelas et al. 2020; Ledoux et al. 2018) or equality of treatment (Friðriksdóttir 2017)).

Third-country nationals who have a salaried or independent worker residence permit depend on their employment or independent activity to enter and reside in the territory of the host Member State. According to the numbers of first-time residence permits for remunerated activities issued by the EU-27 (737,482 in 2016, 905,331 in 2017 and 777,701 in 2018), they represented almost a third of the total number of the first-time residence permits issued during these years (29.6% in 2016, 33.6% in 2017 and 28.6% in 2018), which shows their importance (Eurostat 2020b). The residence permit allows third-country nationals to travel and stay in the other EU Member States. Without employment or independent activity, these third-country nationals not only have their mobility restricted, but when the condition sine qua non of their residence permit disappears, they are obliged to leave the host country.

The Covid-19 pandemic and its negative effects on economies, borders and mobilities, which are still ongoing, can be seen as having an especially high impact on the restricted intra-EU mobility of TCNs as well as on their status in the EU Member States. The closure of borders, together with the economic crisis and the mobility restrictions with which third-country nationals are confronted, puts them in a precarious situation as there is a fear for their employments or independent activities making uncertain their reason for staying and residing in the host country, thus obliging them to leave it. However, this possibility becomes even more complex as the internal and external borders are closed, obstructing the possibility of making secondary moves to find employment or preventing a return to their country of origin. This situation can result in these third-country nationals falling into an irregular and thus precarious residency situation. The pandemic is primarily a health crisis but in the migration context it becomes also a humanitarian and an economic crisis, one which demonstrates the difficulties Member States have in responding to the situation through ad-hoc legal and policy interventions. So far, few scientific articles have dealt with Covid-19 and its impact on labour migration (i.e. Papademetriou and Hooper 2020; van Barneveld et al. 2020).

This paper analyses the different measures taken by EU Member States and Norway regarding third-country nationals during the Covid-19 pandemic specifically dealing with: a) border closures; b) operation of migrations services, c) granting a tolerated stay to temporary staying migrants and those whose residence permits were expiring; d) unemployment and its impact on their right to stay: and e) how Luxembourg as a case study dealt with the legal situation of third country nationals in its territory. Luxembourg was chosen as it is the centre of the largest cross-border region in the EU which has been impacted by the measures taken by the neighbouring countries. This paper will show that the actions were taken without a contingency plan and without any coordination between Member States.

## Methodology

The Covid-19 pandemic crisis forced the European Union and its EU Member States to close the external and internal borders. To obtain up-to-date, reliable and comparable information, the European Migration Network (EMN) decided to use its ad-hoc query system to collect this information. The ad-hoc query system is an internal electronic system used by the EMN national contact points (EMN NCPs) which allows an EU Member State to request detailed information on a migration or asylum issue, giving the other EMN NCPs a deadline of 4 weeks or less (depending on the urgency) to answer. The European Commission jointly with EMN Luxembourg launched four ad-hoc queries to the all NCPs between the 8th and 23th April 2020. The first ad-hoc query, entitled “Measures taken in the field of legal migration as a result of the Covid-19”, was answered and updated by 25 EU Member States (Austria (which has to be excluded from this publication as its responses were for internal use only), Belgium, Bulgaria, Cyprus, Croatia, Czech Republic, Estonia, Finland, France, Germany, Greece, Hungary, Ireland, Italy, Latvia, Lithuania, Luxembourg, Malta, Netherlands, Poland, Portugal, Slovakia, Slovenia, Spain, Sweden) and Norway. The second ad-hoc query, entitled “Measures taken in the field of acquisition of citizenship as a result of the Covid-19 crisis”, was responded to and updated by 25 EU Member States (see above) and Norway. The third ad-hoc query, entitled “Seasonal Workers during the Covid-19 pandemic crisis”, was answered and updated by 24 EU Member States (Belgium, Bulgaria, Cyprus, Croatia, Czech Republic, Estonia, Finland, France, Germany, Greece, Hungary, Ireland, Italy, Latvia, Lithuania, Luxembourg, Malta, Netherlands, Poland, Portugal, Slovakia, Slovenia, Spain, Sweden) and Norway, and the fourth ad-hoc query, entitled “Covid-19 pandemic crisis and unemployment of TCNs”, was answered and updated by 25 EU Member States (see above) and Norway. This information allows the creation of two matrices (the first including the answers to the first two ad-hoc queries and the second using the information related to the last two ad-hoc queries mentioned above), using Excel spreadsheets to process the information. These matrices were shared with the EU Member States to update the information on a weekly basis, and were updated weekly until 15th June 2020. Moreover, we have analysed legal documents in Luxembourg that were affecting the situation of third-country nationals in the first 8 months of 2020.

## The Covid-19 pandemic and the European borders

On 31st December 2019, the WHO obtained information that cases of ‘viral pneumonia’ were reported in Wuhan, People’s Republic of China (WHO 2020b). On 5 January 2020, the WHO issued the first Disease Outbreak News Report on Covid-19. At that point in time, the WHO advise against the imposition of any travel or trade restrictions on China (WHO 2020a). At the time, the picture looked like isolated cases that could be contained inside China. As there was very little information on the virus, there was no immediate response from the European Union on how to prepare for it. When the Chinese authorities decreed a lockdown of the city of Wuhan and other cities in the province of Hubei on 23rd January 2020 to contain the spread, European countries and others did not prepare adequately to deal with the prospect of a pandemic, forgetting that in a globalised world, people travel from and to Europe. Nevertheless, airlines began suspending long-haul flights from China into the EU, beginning with Air France on 30th January 2020. It was only on 31st January 2020 that the Italian government announced it was suspending visas and all flights between Italy and China, and declared a state of emergency in the country after doctors confirmed two Chinese tourists in Rome had tested positive for the coronavirus (Schengenvisainfo News 2020a, 2020b, January 31). However, as this was a unilateral move, what happened was that Chinese tourists and Italian nationals returning from China continued to enter Italy by triangulating with other airports in the European Union. On 4th February 2020, France issued a warning against any non-essential travel to China and suggested that all of its citizens should leave China (Ministère des Affaires étrangères 2020).

In the first few days of February 2020, some EU Member States began to question whether they should suspend the granting of visas to Chinese individuals coming to the European Union for business and tourism purposes. Some EU Member States were still granting visas while others had suspended the procedure as a whole (European Migration Network 2020a). When the first cases of the virus began to be detected in Europe, there were unilateral responses by EU Member States to close their internal borders, beginning with Austria on 11th March 2020 (European Commission, Migration and Home Affairs 2020) and continuing until 16th July 2020. It was not until 17th March 2020 that the European Union decided to close the external borders of the Schengen area. This shows one more the complex and incomplete entity of Schengen, as it continues to struggle with overcoming the notion of national suzerainty of the Member States (Pascouau 2016), which was demonstrated as some EU Member States continue to close their internal. Even though a virus does not identify and respect borders, Covid-19 has had a direct impact on border management for the European Union, not only at the external borders, but also at the internal borders, almost annihilating the Schengen acquis overnight. The pandemic crisis has had a negative impact in most of the European economies, throwing the European Union into the deepest recession since the end of World War II, to the point that during the first 6 months of 2020, most European economies have been working at between 25% and 30% of their capacity and GDP has contracted by 3.2%. EU Member States have been forced to take fiscal and monetary measures to support the recovery and stabilize the economy amid rising unemployment (OECD 2020). The risk surrounding the growth projections depends on the second wave of infections that could trigger new national lockdowns (Eurostat 2020a). Even though, the unemployment has been declining in the EU since 2013 (11,4%), being 8,1% in 2017, 7,2% in 2018 and having reached 6,7% at the end of 2019 (Eurostat 2020d), Eurostat estimates an unemployment rate of 7.5% as at the end of December 2020 which represents 16 million unemployed persons (Eurostat 2020c).

## Operational measures taken to deal with migration issues

This unforeseen situation brought restrictions on working arrangements and travel, due to the implementation of measures taken to contain the Covid-19 pandemic, which has had an impact on all areas of the economy and public services, including on immigration services. With the closure of public offices and the restrictions on travel necessitated by the pandemic, there has been a consequent impact on the processing, renewal and validity of temporary authorisations of stay, residence permits and long-stay/short-stay visas for third-country nationals in the EU Member States and Norway (Sheridan and Sommarribas 2020).

Implementation of these measures was however asymmetrical between the EU Member States. At the consular level abroad, the functioning of the long-term visas was disrupted because there was a partial or full closure of the immigration services in the Member State (see below), but also because the host country applied lockdown measures which affect the functioning of the consular services. An additional factor was that Covid-19 has completely disrupted any type of travel between third countries and the European Union, which in some cases rendered the granting of visas useless, as the individual could not enter the EU. This generated a situation where some EU Member States reported introducing restrictions on the processing of visas, temporary authorisations of stay and residence permits (Cyprus, Croatia, France, Italy, Lithuania, Slovakia, Slovenia, Poland and Norway). Concerning immigration services, most EU Member States and Norway reported restrictions on direct interaction with customers, except for Sweden. Sweden has not introduced any measures as yet, as the examination of applications for residence permits is based on laws with which the Swedish Migration Agency must comply, and since the laws do not allow for any exceptions due to extraordinary events, the procedures have to be respected. In the rest of the EU Member States, immigration services suffered partial or full closures. In the ones that maintained a partial service, the enforcement of public health recommendations was applied (i.e. use of masks, social distancing, a maximum number of clients at the same time, use of a strict appointment system). The other EU Member States, which implemented full closure to the public, tried to facilitate access for clients via an online platform or through electronic means (i.e. e-mails). A good example is Estonia, which has an electronic migration management system. In some cases, as in Ireland, the applications can be submitted via e-mail with a copy of the scanned documents, but in due course the originals will have to be presented. Most EU Member States implemented remote working when possible and maintain an appointment system at service points to deal with urgent cases (i.e. to issue a residence permit) (e.g. Cyprus, Croatia, Czech Republic, Finland, Luxembourg, Netherlands, Poland, Portugal and Slovakia). Also, the processing of visas, temporary authorisations of stay and residence permits has been prioritised for certain categories of third-country nationals (i.e. family members of a legally resident third-country national, healthcare and eldercare professionals, diplomats, workers engaged in the transport of goods, police officers, protection teams and humanitarian workers) (Sheridan and Sommarribas 2020). Regarding the processing of applications, there were different approaches. Some EU Member States continued processing (Belgium, Croatia, Finland, Greece, Ireland, Netherlands and Poland), others limited the processing to a limited category of applications (France, Latvia, Luxembourg, Malta, Slovakia, Slovenia and Spain) and others suspended the processing of applications during the crisis. Spain extended the validity of residence permits for 6 months after the state of crisis is lifted. Finally, some EU Member States (i.e. Italy) took measures to extend the validity of the foreign documents requested for third-country nationals so that they would not be obliged to have to request them again. The resumption of direct interactive services for clients as the public health situation improved began in mid-May 2020 with the reopening of offices, but in certain cases maintaining an appointment system.

During the second half of 2020, Member States have continued to refine the policies implemented in the early stages of the pandemic in order to keep migration systems functioning and ensure that essential labour market needs are met (European Migration Network 2020b, 2020c) However, the situation at the external borders and internal borders remain uncertain with some Member States closing their external borders (i.e. France and Portugal) and others their internal borders.

## Avoiding that third-country nationals fall in irregular stay situation

In view of the restrictions mentioned above, the immigration authorities of the EU Member States took certain measures to avoid legal migrants falling into an irregular situation. Examples of these measures were: 1) extending the validity of residence permits that expired during the duration of the state of emergency/crisis (i.e. France); 2) extending short-stay visas of third-country nationals who cannot leave the country because of the closure of the internal and external borders; 3) extending the right to stay for people who do not require a visa and who could not leave the territory until the end of the crisis; 4) extending or suspending deadlines or revoking obligations on third-country national during the crisis (i.e. Slovakia) and 5) suspending the deadlines for appeal procedures. However, in some countries such as the Netherlands and Luxembourg, oral hearings were maintained. In Croatia, the extension was of 30 days after the end of the state of crisis. In France, a 180-day extension was granted for all residence permits that expired between 16th March and 15th June 2020. In Ireland, registrations of current valid permits where renewal fell between 20th March 2020 and 20th July 2020 were automatically renewed for a period of 2 months. Luxembourg, for example, extended the validity of the residence permits that expired after 1st March 2020 to 31st August 2020, and extended the right to remain in the territory until 31st July 2020. Malta and Croatia extended the permitted stay of third-country nationals without valid residence permits until those people could actually leave the territory. Nevertheless, there was no uniformity between the EU Member States regarding the measures taken, and the duration of extensions varied between the EU Member States. Other measures that were taken regarding residence permits were designed to address labour market needs, especially regarding healthcare and seasonal work. During the state of crisis, the immigration services continued handling applications from healthcare and eldercare workers, and in some countries for seasonal workers. Even though Sweden did not go into lockdown, it took some measures regarding the needs of the healthcare and agricultural sectors (i.e. extending the duration of stay for the seasonal workers already in their territory). Spain put into place a special protocol regarding healthcare workers which ended on 14th May 2020. Examples of the measures taken to address the shortages of seasonal workers include: 1) extending the validity of residence permits or periods of authorisation to stay for seasonal workers already in the EU Member States (i.e. Estonia, France, Greece, Italy, Slovenia, Spain and Norway); 2) allowing a change of status, profession or sector of third-country nationals to seasonal workers (i.e. Estonia and Poland); 3) specific quarantine rules for seasonal workers; 4) facilitation of entry of seasonal workers only by aeroplane (i.e. Germany) or by specific land borders (i.e. Hungary). In Norway, a new temporary regulation was introduced in March 2020, valid until 31st December 2020, which allows anyone who was in Norway during the coronavirus outbreak in March 2020 with a valid seasonal work permit and who wanted to engage in seasonal work beyond 6 months to apply for a renewed seasonal work permit.

## Unemployment and its impact on residence permits

The Covid-19 crisis is not only a public health crisis; the economic impact on the world economy is the deepest contraction since World War II. According to the European Commission Economic Forecast (Ajean 2020), the EU economy is forecast to contract by 8.3% in 2020 before recovering partially (by 5.8%) in 2021. However, the impact is highly asymmetrical across the EU Member States and industries, as there is the sharpest decline in trade, transport, accommodation and food services as well as arts, entertainment and other services (Ajean 2020). This contraction of the European economies has put most of them in recession. As the economy contracts, a significant number of companies will file for bankruptcy and others will try to reduce the number of employees to surf the crisis. Unemployment reached 7.1% in the EU-27 territory and 7.8% in the Eurozone in June 2020 (Eurostat 2020c). The only country in which the unemployment rate remained stable during the Covid-19 crisis was Germany (Nienaber 2020). Unemployment has a direct impact not only on EU citizens but also on legally resident third-country nationals. The main problem is that all the residence permits of economic legal migrants are conditional on underlying employment (work contract). If the employment relationship disappears there is no valid reason for maintaining the residence permit and this leads to procedures for withdrawal of the residence permit (Sheridan and Sommarribas 2020). In times of crisis and in view of the fact that the proportion of migrant workers amongst low-skilled workers is high, they are more at risk of losing their employment (Fasani and Mazza 2020) because they are “vulnerable to forced shutdowns being frequently employed in non-teleworkable occupations and on [fixed-term] contracts” (Fasani and Mazza 2020). During the lockdown, the EU Member States have taken general support measures to avoid massive layoffs, from which third-country nationals can benefit (even though some critics say that these measures are just delaying layoffs). EU Member States did not amend national laws regulating the withdrawal of residence permits due to a drop-in income or loss of employment by a third-country national. However, most EU Member States did not immediately start procedures to withdraw residence permits. Most of them allow the residence permit to continue until the expiry date or for a certain fixed period, to allow the third-country national to find new employment. In some of the EU Member States, this period for finding a job was extended (i.e. Cyprus, Finland, Slovakia and Slovenia). In Bulgaria, Croatia, Hungary, Lithuania and Malta, the procedure for withdrawing the permit did start at the moment the third-country national loses his/her job. In practice, flexibility and leniency have been shown if the third-country national finds another job during the withdrawal procedure (i.e. Croatia) or the circumstances related to the pandemic in the loss of employment are taken into consideration (i.e. Lithuania); in another case, a special service has been established within the employment service to find alternative employment for third-country nationals (i.e. Malta).

## Access to employment benefits and renewal of residence permit due to unemployment

In most reporting EU Member States and Norway, third-country nationals who had lost their jobs were entitled to unemployment benefits in the same way as nationals if the applicant fulfilled the criteria. However, the duration of the benefits generally depends on the age of the person and their contributions to the unemployment insurance scheme. It can vary from 90 days in Hungary up to an indefinite period but with a progressive reduction of the benefits (Belgium).

Some EU Member States do not allow the renewal of a residence permit if the third-country national has lost his/her employment (Bulgaria, Croatia, Estonia, Hungary, Latvia, Lithuania, Malta and Slovakia). This is because the normal rules for renewing a residence permit continue to apply, but in practice these EU Member States provide the opportunity to renew it if the person finds a new job. In the other EU Member States, it is possible to renew the residence permit if the third-country national has lost his job (Belgium, Cyprus, Czech Republic, Greece, France, Italy, Luxembourg, Spain and Sweden). However, certain EU Member States impose conditions, such as a) a specific period to find new employment (i.e. Cyprus and Sweden) or b) having a secure means of support during the previous residence permit (i.e. Finland and Portugal). Finally, in the case of a drop in/loss of income (sufficient means of subsistence), there are two positions in the EU Member States:

- restrictive application of the law (Bulgaria, Finland, Netherlands, Slovenia and Sweden), in which if the individual does not reach the salary threshold the residence permit will not be renewed; and
- a flexible approach taking into consideration all the circumstances (Belgium, Cyprus, Croatia, Czech Republic, Estonia, France, Germany, Ireland, Italy, Latvia, Luxembourg, Malta, Portugal, Slovakia and Spain).

## Luxembourg and the pandemic

In the next section, we undertake a more in-depth analysis of Luxembourg as a case study. Like other European countries, Luxembourg had to react quickly to fight the Covid-19 pandemic. Given the geographical location of Luxembourg (landlocked between three neighbouring countries) and its dependence on the border workforce of the Greater Region, Luxembourg had to take legal and diplomatic measures when neighbouring countries started closing and/or controlling intra-European borders, starting with Germany, which closed borders with the Grand Duchy on 16th March 2020 (European Commission, Migration and Home Affairs 2020), notification no. 130). This measure per se had a negative impact on Luxembourg, as a large proportion of the health workers and caregivers working in Luxembourg hospitals are domiciled in Germany. Following negotiations with the German authorities on 19th March 2020, Germany permitted cross-border workers to cross at specific border crossing points but under strict conditions. The situation lasted until midnight on 15th May 2020 (Answer to Parliamentary Question no. 2274 n.d.). France and Belgium had also introduced border controls to avoid unnecessary travel.

On 18th March 2020, Luxembourg declared a state of crisis on the basis of Article 32–4 of the Constitution of the Grand Duchy of Luxembourg (Grand Ducal Regulation 18.03.^2020^). The state of crisis was extended until 24th June 2020 (Law 24.03.^2020^, art. 1). In view of the diversity of the population resident in the Grand Duchy, the Government decided to post information on Covid-19 on the public authorities’ websites in German, English, French and Luxembourgish (Answer to Parliamentary Question no. 2024 n.d.).

The Grand Duchy of Luxembourg took the following measures in regard to labour migration:

In order to prevent the spread of the Covid-19 virus and to protect public health, the Government took the decision to temporarily close the Information Desk of the Foreigners’ Service, but this was replaced by reinforced access to the service via telephone (Answer to Parliamentary Question no. 2041 n.d.). The Registration Desk of the Foreigners’ Service for the collection of biometric data was open to the public by appointment only from 8:30 a.m. to 12 noon and from 1 p.m. to 4 p.m. Monday to Friday (Ministry of Foreign and European Affairs 2020a). The Immigration Directorate had indicated that there was no possibility during lockdown to submit files and documents in person, but that documents could be sent by post (Government of Luxembourg 2020a). However, for duly justified emergencies, reception of the public by appointment was ensured. The appointment was to be made by e-mail, specifying the context of the emergency (EMN Luxembourg 2020a).

During the first phase of the lockdown, no decision was taken in the context of legal migration except in cases of emergency. In general, third-country nationals applying for a residence permit must do so from their country of origin, otherwise the application is declared inadmissible (Amended law of 29.08.^2008^, art. 39). The time limits for taking decisions laid down in the Immigration Law (Amended law of 29.08.^2008^) only start to run when the file is considered to have been completed by the agent handling it. While the closure of the external borders was extended until 31st December 2020 (see below) — except for certain groups of third-country nationals — new residence permits were no longer issued, except in duly justified cases of urgency. At the same time, the registration and issuing of new residence permits is currently limited to justified emergencies (Answer to Parliamentary Question no. 2041 n.d.).

Since 13th May 2020, the reception desks have been reopened but they are only available by appointment via the website of the Directorate of Immigration. However, this possibility exists only for foreigners who have been invited to apply for such appointments. These appointments must be made for the registration and/or issuing of biometric residence permits. The information offices remain closed, and information is given only by telephone and e-mail (Ministry of Foreign and European Affairs 2020b).

The external borders of the Schengen area were closed on 17th March 2020 for a period of 30 days. Consequently, the Luxembourg Government decided that third-country nationals could not enter the territory from 18th March 2020 at 6 p.m. for a renewable period of 1 month (Grand Ducal Regulation 18.03.^2020^, art. 14 (1)). The Grand Ducal Regulation establishes derogations for the following third-country nationals:

- Nationals who hold a long-term residence permit or who have a valid residence permit;
- Health professionals, health researchers and care professionals for the elderly;
- Cross-border workers;
- Persons employed in the freight transport sector and in the transport of goods and passengers, including airline crews;
- Members of the diplomatic corps, personnel of international organisations, military organisations, personnel in the field of development cooperation and humanitarian aid, in the course of their duties;
- Transit passengers;
- Passengers travelling for urgent and duly justified family reasons;
- People wishing to apply for international protection or for other humanitarian reasons.
- Citizens of the European Union, the United Kingdom and Schengen Associated Countries, as well as their family members, have been exempted from these travel restrictions, with the aim of returning to their homes.

With the end of the state of crisis, the Law of 24th June 2020 introducing certain temporary measures relating to the application of the amended Immigration Law extended temporary entry into the territory until 31st December 2020 (Grand Ducal Regulation 11.09.^2020^).

Nevertheless, the Grand Ducal Regulation of 1 July 2020 contains the following categories of TCN for whom the entry ban does not apply:

- Nationals who hold a long-term residence permit or who have a valid residence permit;
- Health professionals, health researchers and care professionals for the elderly;
- Frontier workers;
- Seasonal agricultural workers;
- Persons employed in the freight transport sector and in the transport of goods and passengers, including airline staff;
- Members of the diplomatic corps, staff of international organisations and people invited by such international organisations whose physical presence is required for the proper functioning of those organisations, military personnel, personnel in the field of development cooperation and humanitarian aid, and civil protection personnel in the performance of their respective duties;
- Transit passengers;
- Passengers travelling for urgent and duly justified family reasons;
- Seamen;
- People wishing to apply for international or other humanitarian protection;
- Third-country nationals travelling for the purpose of studies;
- Highly qualified third-country workers if their employment is economically necessary and their work cannot be postponed or carried out from abroad.

The same Grand Ducal Regulation also establishes an initial list of third countries whose nationals may enter the territory of the Grand Duchy. This list has subsequently evolved and at the moment it exempts the nationals of the following countries: Australia, Canada, China (subject to reciprocity at EU level), Georgia, Japan, New Zealand, Rwanda, South Korea, Thailand, Tunisia and Uruguay. Nationals of these countries must be in possession of official documents proving their residence in one of these countries (residence permit, residence certificate, work permit, etc.). These documents must be accompanied by a translation into French, German, Luxembourgish or English. According to the Grand Ducal Regulation of 7th August 2020, any third-country national who is at least 11 years of age in the categories of persons mentioned above and who wishes to travel by air to Luxembourg from a third country which is not on the list of third countries above — even if he has a temporary residence permit or a residence permit — must, before embarking, submit a negative result from a Covid-19 test. The test must have been carried out less than 72 h before the flight (Amended Grand Ducal Regulation 20.06.^2020^, art. 2ter). Aircraft crew members have been exempted from the requirement to present a negative test result, as have third-country nationals in transit, namely passengers on stopovers with a connecting flight who do not leave the transit area at Luxembourg airport (Government of Luxembourg 2020b).

From a health perspective, there have not been any changes in the conditions for entry into the territory. Luxembourg had established a list of risky areas at the beginning of the pandemic; however, the authorities did not introduce a quarantine period for international travellers, with Luxembourg having as its only external border Luxembourg International Airport, which has been closed for passenger transport since 18th March 2020. Once the airport reopens, Luxembourg has the facility for any passenger to be tested on arrival, regardless of where they are flying from. However, quarantine could be ordered by the Health Inspectorate if the individual arriving from abroad showed symptoms of Covid-19 infection.

As regards the labour market test, the deadline for ADEM to examine whether the job vacancy could possibly be filled by a registered jobseeker (Luxembourgish national, EU citizen or TCN who is legally residing in the territory) was increased during the health crisis from three to 6 weeks. If, within a period of 6 weeks from the declaration of a job, ADEM has not offered the employer a candidate who has met the profile required for the declared post, the employer may request a certificate certifying the right to recruit the person of his choice for the post (ADEM 2020).

On 18th March 2020, the Luxembourg Government took the decision to extend the period of validity of visas, temporary residence permits, residence cards and residence permits expiring after 1st March 2020 for the duration of the state of crisis (Government of Luxembourg 2020c). Similarly, third-country nationals not subject to the visa requirement and whose stay has just exceeded 90 days may stay legally for the duration of the state of crisis (Grand Ducal Regulation 18.03.^2020^, art. 13). These measures were applied automatically and did not require any particular steps to be taken by third-country nationals.

With the end of the state of emergency, the following temporary measures were adopted:

For third-country nationals who made their declaration of arrival between 1st January and 31st July 2020: the period within which they must apply for a residence permit to be issued is extended from 3 months to 6 months (Law of 20.06.^2020^, art. 1 (3));

For residence permits issued by Luxembourg which expire after 1st March 2020: the period of validity is extended until 31st August 2020 (Law of 20.06.^2020^, article 1 (2));

For third-country nationals holding a short-stay visa and those not subject to a visa requirement and whose stay has just exceeded 90 days after 1 March 2020, their stay is regularised until 31st July 2020 (Law of 20.06.^2020^, art. 1(3)).

To facilitate their exit from the Schengen area, the people in question could make an appointment with the Bureau des Passports, Visas et Legalisations (BPVL) with a view to having a ‘return visa’ issued.

Another measure taken in the context of the crisis management of Covid-19 was the suspension of deadlines for appeals before administrative courts (Grand Ducal Regulation 25.03.^2020^, art. 1 (2)). The deadline for bringing proceedings before the administrative courts in the first instance are extended (Grand Ducal Regulation 01.04.^2020^) as follows: (a) periods expiring during the state of crisis are extended by 2 months from the date of the end of the crisis; (b) deadlines due within 1 month of the end of the crisis are extended by an additional month from their due date.

Luxembourg did not have to take any measures with regard to the withdrawal of residence permits due to loss of employment. The Immigration Law (Amended law of 29.08.^2008^, art. 43 (4)) envisages that if, at the time of renewal of the residence permit, the salaried worker cannot prove that s/he had actually worked during the period of validity of the residence permit or work permit, or if the renewal occurs during the period when the TCN is receiving unemployment benefits, the residence permit or work permit is renewed for a maximum period of 1 year. Normally, third-country nationals are granted the opportunity to find other employment if they have lost their employment for reasons unrelated to them (as is the case with Covid-19). Under the cover of their residence permits, they may register with the National Employment Agency (Agence pour le développement de l’emploi – ADEM) as jobseekers, which gives them access to help and guidance in their search for a job and a range of services geared specifically towards jobseekers (EMN Luxembourg 2020b). The timeframe for looking for new employment will be for the duration of the residence permit, or will have a deadline of 1 year if it was renewed (see above). Registration with ADEM is a precondition for obtaining a range of monetary benefits paid by ADEM or other organisations. The third-country national has to hold a valid residence permit in order to be eligible for unemployment benefits. In principle, the duration of entitlement to payment of unemployment benefits is equal to the duration of work, in whole months, during the period taken as the reference basis for the calculation. The maximum period is 12 months (there are some exceptions in which it can be extended beyond 12 months) (EMN Luxembourg 2020b).

## Conclusions

The Covid-19 crisis is not only a public health crisis but also an economic crisis that has not previously been seen in the history of Europe. The current economic situation is serious, as the crisis has wiped out 10 years of economic growth in less than 4 months, generating a serious recession due to the contraction of the economy, which will have a significant impact on unemployment. The crisis has shown not only that the European Union as well as the EU Member States did not have contingency plans to deal with a crisis of this nature and they were taken by surprise. Even though there could have been learning processes after the financial crisis or the closing of borders due to terrorism or the 2015 “migration crisis”, the EU countries were developing national strategies to deal with and to secure a third-country national workforce in their countries – especially in sectors of the labour market that were declared to be of systemic relevance.

The crisis not only has demonstrated that there has not been a uniform approach from the European Union and its Member States in the migration context but has also shown that the individual actions of Member States demonstrate the influence of national sovereignty. The benefit of a uniform approach will allow Member States to develop synergies and avoid duplication and manage migration in a rational way through the exchange of information and good practices. The challenge on the other hand, is the different level of economic development and the medical infrastructure to fight the virus in the Member States, which make difficult to implement homogenic solutions to deal with economic paralysis and unemployment.

The current situation has demonstrated that most EU Member States continue to depend on physical files in the migration management process in order to take decisions, which will subsequently generate a delay in decisions being taken. Member States have been implementing online solutions as a reaction to the crisis. However, in the medium term, EU Member States will have to provide for the implementation of an electronic migration management system which allows the customer to apply online and the responsible agent to have access to that electronic file online, allowing remote connection if necessary. This can be seen as an opportunity, as it allows the possibility of implementing a complete electronic migration management system, which will simplify the handling of applications and reduce the backlog in the immigration services whilst simultaneously keeping the staff and applicants out of danger.

During the lockdown, EU Member States have demonstrated flexibility, leniency and understanding with regard to the residence permits, temporary authorisations of stay and long-term/short-term visas, to prevent some third-country nationals from falling into irregular situations because of “force majeure”. Member States have implemented support measures to mitigate the negative effect of the pandemic with regard to enterprises and employees. These are general measures which also benefit third-country nationals. Third-country nationals who lost their employment have access to unemployment benefits just as nationals do.

Nevertheless, the national laws regulating the withdrawal of residence permits for loss of employment have not been amended, so third-country nationals who lose their jobs risk the possible loss of their residence permits and thus becoming subject to a return decision. It is clear that third-country nationals are highly vulnerable, as they represent a high proportion of the low-skilled workforce, which is hired on temporary contracts and in employment which is not teleworkable, and in consequence they are subject to loss of their employment if a shutdown of the firm happens. Most EU Member States have demonstrated leniency and flexibility in the withdrawal and renewal of residence permits if the third-country national can find new employment. However, it is clear that in a recession, finding new employment becomes a difficult issue and it is probable that due to the scarcity of available work, third-country nationals will be competing with EU citizens for the same positions, which will make their stay in the European Union more difficult.

The case of Luxembourg shows clearly how a country depends on decisions made by neighbouring countries, as the closure of the borders carried out by Germany, France and Belgium forced Luxembourg to find internal solutions.

Goldner Lang's (2018) “desirable migrants” are in this case the systemically relevant workforce, but on the other hand measurements were taken for all third-country nationals living in the countries. At the same time, “spatial temporal waiting zones” (Della Torre and De Lange 2018) have been extended to all people worldwide, regardless of whether they are third-country nationals or not. However, the long-term effects on third-country nationals will depend greatly on their status and on the need for a decreasing labour market to retain their professions.

The crisis is not over and most of the issues that are analysed in this article may change in the next few months, depending on the development of the pandemic and the policies implemented by Member States.

Moreover, further research is needed from i.e. economic, psychological, social and political perspectives on what influences these measures have had on the third-country nationals themselves, on the labour markets and also on longer-term political and legal measures.
